# High-dose methotrexate is effective for prevention of isolated CNS relapse in diffuse large B cell lymphoma

**DOI:** 10.1038/s41408-021-00535-y

**Published:** 2021-08-12

**Authors:** Shin Yeu Ong, Sanjay de Mel, Nicholas Francis Grigoropoulos, Yunxin Chen, Yan Chin Tan, Melinda Si Yun Tan, Lawrence Cheng Kiat Ng, Yuh Shan Lee, Colin Phipps, Yeow Tee Goh, Kar Ying Yong, Xin Liu, Wee Joo Chng, Soon Thye Lim, Chandramouli Nagarajan

**Affiliations:** 1 Department of Haematology, Singapore General Hospital, Singapore, Singapore; 2 Department of Haematology-Oncology, National University Cancer Institute Singapore, National University Health System, Singapore, Singapore; 3 Parkway Cancer Center, Singapore, Singapore; 4 NUS Center for Cancer Research and Dept of Medicine, Yong Loo Lin School of Medicine, NUS, Singapore, Singapore; 5 Cancer Science Institute of Singapore, NUS, Singapore, Singapore; 6 Division of Medical Oncology, National Cancer Centre, Singapore, Singapore; 7 Duke-NUS Medical School, Singapore, Singapore

**Keywords:** B-cell lymphoma, Preventive medicine

## Abstract

The role of central nervous system (CNS) prophylaxis with high-dose methotrexate (HDMTX) in DLBCL is controversial. In this retrospective study, we evaluated the efficacy of prophylactic HDMTX on isolated CNS relapse, concomitant CNS and systemic relapse, systemic relapse, and survival outcomes in 226 patients with newly diagnosed DLBCL and high-risk CNS International Prognostic Index (CNS-IPI) score treated with RCHOP. The three-year risk of isolated CNS relapse was significantly lower in patients who received HDMTX, at 3.1% compared to 14.6% (*P* = 0.032) in those who did not. However, neither concomitant CNS-systemic relapse rates, systemic relapse rates, nor three-year PFS and OS were significantly different between treatment groups in multivariable analysis. Among propensity score-matched patients (*N* = 102), HDMTX was also associated with significantly lower isolated CNS relapse rates (HR 0.06, 95% CI 0.004–0.946, *P* = 0.046). HDMTX was well tolerated with manageable toxicities when given at a dose of 3 g/m^2^ by day 3 of RCHOP chemotherapy. Using propensity score matching and multivariable regression to yield treatment groups with well-balanced covariates, we showed that prophylactic HDMTX improved isolated CNS relapse rates but did not decrease concomitant CNS-systemic relapse rates, systemic relapse rates, or improve survival outcomes.

## Introduction

Central nervous system (CNS) relapse occurs in 1–14% of patients with diffuse large B cell lymphoma (DLBCL) and carries a dismal prognosis with survival of 2–5 months [1]. With concerns around the effectiveness of intrathecal (IT) prophylaxis which may not reach measurable concentrations in the brain parenchyma [2, 3], systemic high-dose methotrexate (HDMTX) has been recommended by some guidelines for patients at high risk of CNS relapse [4, 5]. However, evidence supporting this practice is limited to small retrospective studies [6, 7], and recent studies demonstrate no benefit of HDMTX in preventing CNS relapse [8, 9]. Given the potential for HDMTX to be associated with significant toxicity and delays of RCHOP chemotherapy which may compromise systemic control [10], further evaluation of the role of HDMTX in mitigating the risk of CNS recurrence is warranted.

There is ongoing debate on the utility of HDMTX in preventing CNS relapse in view of heterogeneity of existing literature without definitive randomized controlled trials to base practice. Importantly, current studies assessing the utility of HDMTX do not differentiate isolated CNS relapse from concomitant CNS and systemic relapse when evaluating the prophylactic effect of HDMTX. While isolated CNS relapse may be prevented by effective CNS prophylaxis, concomitant systemic and CNS relapse likely represents failure of systemic treatment with subsequent acquisition of CNS-penetrating subtypes of malignant clones [11–13]. Puckrin et al. recently showed lack of efficacy of HDMTX after adjusting for confounding factors using propensity score matching analysis, but the majority (59%) of patients with CNS relapse had concurrent systemic disease. Ideally a randomized controlled trial will be best suited to answer the question about efficacy of prophylaxis, but the rarity of CNS relapse is a major barrier to conducting adequately powered clinical trials. In addition, several retrospective studies suggest that HDMTX may be beneficial for systemic disease, based on improved PFS and OS without improvement in CNS relapse rates, but did not differentiate between isolated versus concurrent CNS relapse [14, 15].

Given these questions about efficacy of HDMTX for CNS versus systemic relapse, we sought to determine if HDMTX reduced rates of isolated CNS relapse, concomitant CNS-systemic relapse, or systemic relapse in a group of patients at high risk for CNS relapse from three tertiary institutions in Singapore, compared to a closely matched historical cohort, including a propensity score matching analysis to assure comparability. We also determined the rate of toxicities involved in prophylaxis.

## Subjects and methods

We conducted retrospective chart reviews of patients with DLBCL treated with RCHOP chemotherapy at three academic medical centers in Singapore, between January 2016 and December 2018 for patients with a confirmed diagnosis of DLBCL by WHO criteria. Patients with high-grade transformation of low-grade lymphoma, HIV-associated DLBCL, Burkitt lymphoma or DLBCL with CNS involvement at diagnosis were excluded. Anonymized data collection was granted a waiver of consent with deidentification by the Institutional Review Board. Since 2016, CNS prophylaxis with HDMTX at doses of at least 1 g/m^2^ either intercalated with RCHOP or after six cycles of RCHOP was recommended for patients at high risk of CNS relapse, namely CNS-IPI 4–6, or specific anatomical site involvement (breast, testis, kidney/adrenal). Patients receiving additional IT prophylaxis were not excluded.

This group was compared with a historical cohort of DLBCL patients who had high CNS-IPI score treated between 2005 and 2016 with RCHOP applying the same exclusion criteria. CNS relapses were diagnosed by cerebrospinal fluid cytology or flow cytometry, or brain/vitreous biopsy. The primary endpoints were time to CNS disease (isolated or concomitant systemic disease) and systemic relapse measured from the start date of 1^st^ cycle of chemotherapy. PFS was defined as time from start date of chemotherapy to date of disease progression or death from any cause and overall survival was defined as time from start date of chemotherapy to date of death from any cause or date of last follow-up in surviving patients. Probabilities of time to CNS or systemic disease were estimated using the Kaplan–Meier method, and survival curves were compared between groups using the log-rank test. Three-year estimates of the risk of CNS (isolated or concomitant with systemic disease) and systemic relapse were reported with 95% confidence intervals (CIs). Univariable and multivariable analyses for time to CNS or systemic relapse, PFS and OS were performed using the Cox proportional hazards regression method. In view of the low number of outcome events, we used a stepwise approach to select confounding factors into the multivariable model, including statistically significant variables (*P* < 0.1) in the univariable analysis. Propensity score matching with the nearest neighbor method with caliper width of 0.2 was used to match patients treated with HDMTX to those treated without. A *P* value <0.05 was considered statistically significant. Adverse events were graded according to the Common Terminology Criteria for Adverse Events version 5.0. Statistical analyses were performed using Stata version (StataCorp, College station, TX).

## Results

### Patient characteristics

Baseline characteristics of all 226 patients are summarized in Table 1, with further stratification by isolated CNS relapse, concomitant CNS and systemic relapse, and systemic relapse. Median follow-up was 20 months for the whole cohort (range 10 months to 8 years) and 2.3 years (range 12 months to 8 years) for surviving patients. The median age was 65 years, with a slight male predominance. 84% had two or more extranodal site involvement and 85% had a high CNS-IPI score. Only 4.4% had double hit lymphoma.

**Table 1:** Baseline patient characteristics.

	No relapse (*n* = 149)	CNS only recurrence (*n* = 24)	Systemic only recurrence (*n* = 43)	Concurrent recurrence (*n* = 10)	*P*^a^
Age, mean (SD), *y*	62.5 ± 11.0	63.1 ± 12.7	63.3 ± 10.7	65.7 ± 7.0	0.830
Male	77 (52%)	16 (67%)	23 (54%)	4 (40%)	0.460
Stage 3 or 4	137 (92%)	23 (96%)	43 (100%)	10 (100%)	0.420
Elevated LDH	133 (89%)	21 (88%)	39 (91%)	10 (100%)	0.714
EN site ≥1	117 (79%)	22 (92%)	41 (95%)	10 (100%)	0.015
EN site involvement					
Bone marrow	52 (34.9%)	16 (66.7%)	17 (39.5%)	6 (60%)	0.016
Kidney/adrenal	37 (24.8%)	10 (41.7%)	7 (16.3%)	4 (40%)	0.096
Testis	1 (0.7%)	1 (4.2%)	1 (2.3%)	3 (30%)	<0.001
Breast	1 (0.7%)	2 (8.4%)	1 (2.3%)	0 (0%)	0.065
Double hit	8 (5.3%)	1 (4.2%)	1 (2.3%)	0 (0%)	0.746
Cell-of-origin (GCB)	53 (36%)	12 (50%)	13 (30%)	5 (50%)	0.328
CNS-IPI					
Low	10 (6.7%)	1 (4.2%)	0 (0%)	0 (0%)	0.298
Intermediate	19 (12.8%)	1 (4.2%)	4 (9.3%)	0 (0%)	
High	120 (80.5%)	22 (91.7%)	39 (90.7%)	10 (100%)	
IPI					
1–2	26 (17.5%)	22 (91.7%)	42 (97.7%)	0 (0%)	0.061
3–5	123 (82.6%)	2 (8.3%)	1 ((4.2%)	10 (100%)	
IT methotrexate	27 (18.1%)	4 (16.7%)	5 (11.6%)	3 (30%)	0.537
HDMTX	53 (35.6%)	2 (8.3%)	10 (23.3%)	1 (10%)	0.015

Data are *n* (%), unless otherwise stated.

^a^Clinical characteristics between the groups were compared using the *χ*^2^ test for categorical variables and one-way ANOVA for continuous variables. *LDH* lactate dehydrogenase, *GCB* germinal center B-cell like, *EN* extranodal, *IT* intrathecal, *HDMTX* high-dose methotrexate.

### High-dose methotrexate prophylaxis

Prophylactic HDMTX was administered to 66 (29.2%) patients. The administered HDMTX dose was ≥3 g/m^2^ in 81% of cycles. HDMTX was intercalated between R CHOP cycles in 52 (79%) patients and delivered at end of treatment in 14 (21%) patients. Of the 226 patients, 85% had high CNS-IPI score 4–6, and there was no significant difference in baseline CNS-IPI score between patients who received versus did not receive HDMTX. Patients who received HDMTX tended to be younger, have more extranodal involvement, as well as lymphoma involving the breast (Table 2).

**Table 2:** Baseline patient characteristics by treatment group.

	No HDMTX (*n* = 160)	HDMTX (*n* = 66)	*P*
Age, mean (SD), *y*	64.2 ± 10.5	59.8 ± 11.4	**0.006**
Male	86 (53.8%)	34 (51.5%)	0.760
Elevated LDH	142 (88.8%)	61(92.4%)	0.406
Stage 3 or 4	153 (95.6)	59 (89.4)	0.077
EN site ≥1	96 (60%)	61 (92%)	**<0.001**
EN site involvement			
Bone marrow	65 (40.6%)	26 (39.4%)	0.862
Kidney/adrenal	44 (27.5%)	14 (21.2%)	0.325
Testis	4 (2.5%)	2 (3.0%)	0.822
Breast	1 (0.6%)	3 (4.5%)	**0.042**
CNS-IPI			0.277
Low	6 (3.8%)	5 (7.6%)	
Intermediate	15 (9.4%)	9 (13.6%)	
High	139 (86.9%)	52 (78.8)	
Double hit	7 (4.4%)	3 (4.5%)	0.955

Data are *n* (%), unless otherwise stated. *LDH* lactate dehydrogenase, *EN* extranodal, *IT* intrathecal, *HDMTX* high-dose methotrexate.

### CNS relapse

There were 24 isolated CNS relapses, 10 concomitant CNS-systemic relapses and 43 systemic relapses. The median time to isolated CNS relapse was 7 months (range 4–50 months), concomitant CNS and systemic relapse was 8 months (range 4–80 months), and systemic relapse/progression was 10 months (range 5–110 months). Most of the CNS relapses, as expected in the rituximab era, were parenchymal (68.2%) or combination of parenchymal and leptomeningeal (13.6%). Median OS after CNS relapse (isolated and concomitant systemic) was 63 days (95% CI 33 to 174 days), and 23 patients (68%) passed away. Median time to death after systemic relapse was 121 days (95% CI 44 to 303 days), and 22 patients passed away (65%). The 2-year isolated CNS relapse risk was 12.9% (95% CI 8.6–19.2), similar to initial CNS-IPI study [1]. CNS-IPI and IPI scores were similar among the groups, but patients with CNS recurrence had more extranodal, bone marrow [16], and testicular involvement (Table 1). Two patients experienced isolated CNS relapse after HDMTX, one of whom received HDMTX at <3 g/m^2^, while the other received 3 g/m^2^ but had an isolated intraocular relapse. Most patients received two HDMTX cycles (range 1–6).

Univariable analysis of risk factors for CNS (isolated and concomitant) and systemic relapse versus no relapse was carried out (Supplementary Table 1). Significant risk factors for CNS relapse (isolated and concomitant) by univariable analysis were involvement of the bone marrow (HR 3.23, 95% CI 1.60–6.54, *P* = 0.001), breast (HR 7.88, 95% CI 1.87–33.17, *P* = 0.005), and testis (HR 5.15, 95% CI 1.81–14.69, *P* = 0.002), while HDMTX prophylaxis reduced the risk (HR 0.22, 95% CI 0.07–0.72, *P* = 0.012). Given that the association between bone marrow involvement and CNS relapse is controversial, we further performed multivariable analysis using a Cox proportional hazards model to assess the independent effect of bone marrow involvement (*n* = 91) on isolated CNS relapse controlling for advanced stage III/IV and high IPI score 3–5. Bone marrow involvement remained a significant risk factor for CNS relapse (HR 3.45 95% CI 1.41–8.45, *P* = 0.007). The possible increased risk of CNS relapse in patients with bone marrow involvement requires prospective validation.

**Fig. 1:**
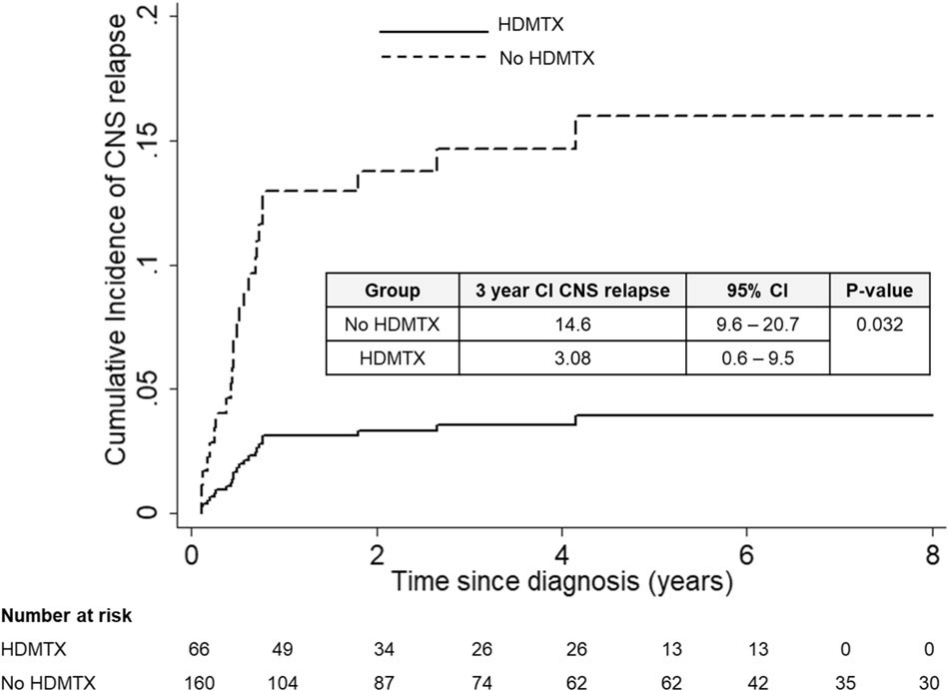
Cumulative incidence of isolated CNS relapse. Cumulative incidence of isolated CNS relapse in the overall population. HDMTX, high dose methotrexate.

For systemic relapse, there was a borderline association with high IPI score 3–5 (*P* = 0.061) and involvement of more than one extranodal site (*P* = 0.081*)*. On multivariable analysis adjusting for competing risk of systemic relapse and death, HDMTX significantly reduced risk of isolated CNS relapse by 84% (HR 0.16, 95% CI 0.03–0.91, *P* = 0.039) (Table 3). The isolated CNS relapse rate was lower in patients who received HDMTX compared to patients who did not receive HDMTX, with a 3-year cumulative incidence of 3.08% (95% CI 0.58–9.51) and 14.6% (95% CI 9.6–20.7) (*P* = 0.032; Fig. 1). However, the 3-year risk of systemic relapse did not differ significantly between the two groups. Similarly, the risk of concomitant CNS and systemic relapse was also not significantly associated with HDMTX (HR 0.26, 95% CI 0.04–2.45, *P* = 0.27) in multivariable analysis.

**Table 3:** Multivariable analysis and propensity score matching based on HDMTX prophylaxis for time to isolated CNS relapse, systemic relapse, PFS, and OS.

	Multivariable analysis		Propensity score matching	
	HR (85% CI)	*P*	HR (95% CI)	*P*
Time to isolated CNS relapse^a^	0.16 (0.03–0.91)	0.039	0.06 (0.004–0.946)	0.046
Time to systemic relapse^b^	1.02 (0.50–2.06)	0.958	3.59 (0.69–18.74)	0.129
PFS^c^	0.60 (0.20–1.12)	0.106	1.12 (0.43–2.92)	0.818
OS^d^	0.61 (0.32–1.17)	0.135	0.63 (0.25–1.61)	0.332

Multivariable model including all factors with *P* < 0.1 from the univariate analysis.

^a^Adjusted for CNS-IPI score 4–6 vs 0–3, extranodal site involvement >1, high risk site involvement (bone marrow, breast, and testis).

^b^Adjusted for IPI score 3–5 vs 0–2 and extranodal site involvement >1.

^c^Adjusted for IPI score 3–5 vs 0–2, extranodal site involvement>1, high risk site involvement (bone marrow, breast, and testis).

^d^Adjusted for IPI score 3–5.

*HR* hazard ratio, *CI* confidence interval, *PFS* progression free survival, *OS* overall survival.

### Survival outcomes

**Fig. 2:**
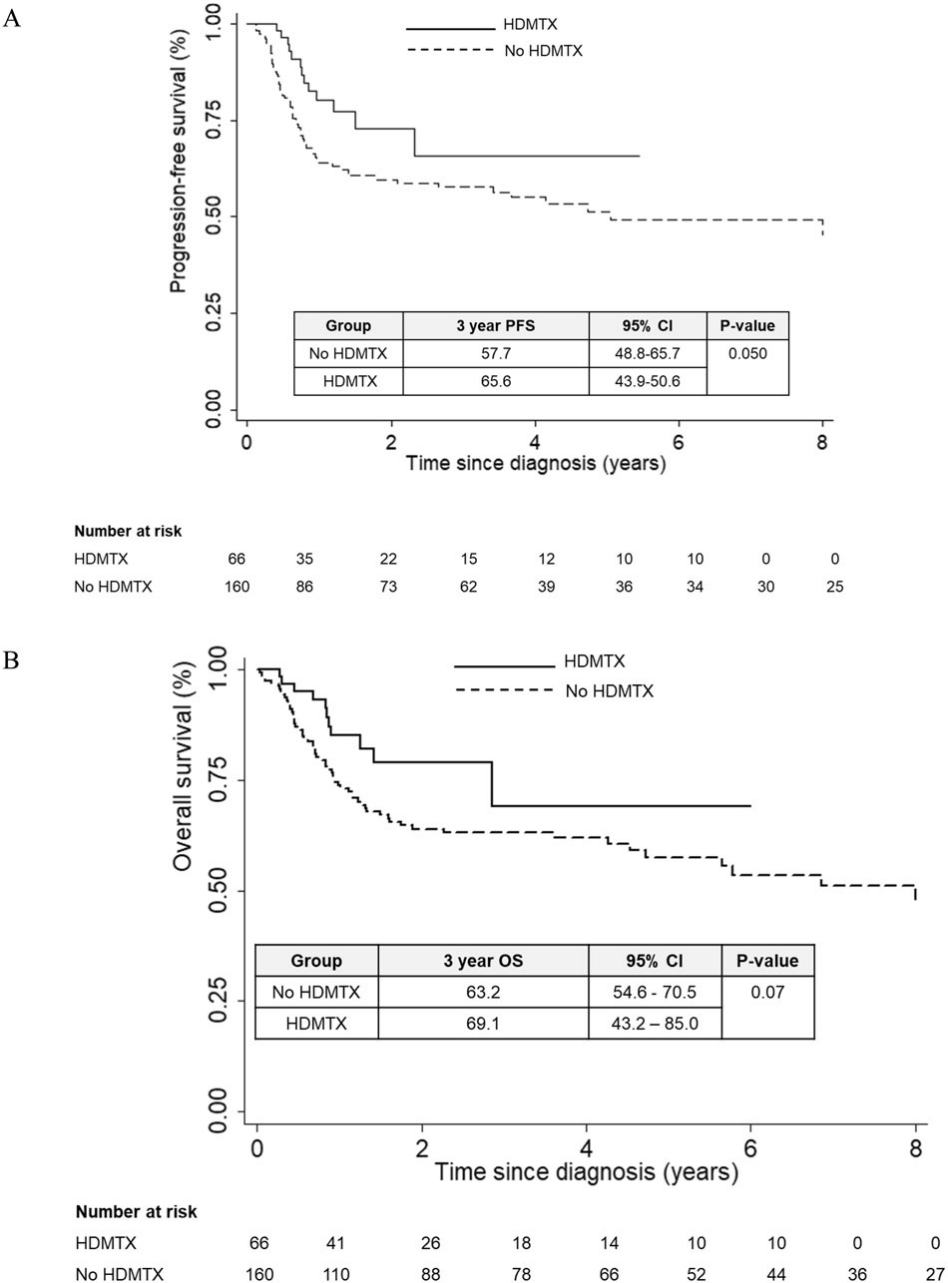
Survival outcomes in the overall patient population. **A** Progression Free Survival by HDMTX prophylaxis. **B** Overall Survival by HDMTX prophylaxis. HDMTX, high dose methotrexate.

In the overall population, the 3-year PFS was 66% (95% CI 44–51) for patients who received HDMTX vs 58% (95% CI 49–66) for patients who did not receive HDMTX, *P* = 0.05. The 3-year OS was 69.1% (95% CI 43.2–85) and 63.2% (95% CI 54.6–79.5), *P* = 0.07 for patients who received and did not receive HDMTX respectively (Fig. 2A, B). In patients with isolated CNS relapse, 2-year PFS was 8.3% (95% CI 1.4–23.3) and 2-year OS was 42% (95% CI 21.9–61.3). In patients with concomitant CNS-systemic relapse, 2-year PFS was 16.7% (95% CI 8.7–31.4) and 2-year OS was 36.2% (95% CI 16.7–56.1). In patients with systemic relapse, 2-year PFS was 16.3% (95% CI 7.2–28.7) and 2-year OS was 53.7% (95% CI 36.8–67.9). Univariable analysis identified high CNS-IPI score, involvement of more than one extranodal site as well as high-risk sites (breast, testis, bone marrow) as significant prognostic factors for PFS. For OS, univariable analysis identified high IPI score as prognostic. HDMTX did not remain an independent prognostic factor for PFS or OS in multivariable analyses (Table 3).

### Propensity score-matched analysis

A total of 51 patients in each group were matched in a propensity score-matched analysis. Baseline characteristics were well balanced between patients receiving HDMTX and without in the matched population (Supplementary Table 2). Among propensity-score matched patients, in a multivariable regression model controlling for high CNS-IPI score, extranodal site involvement >1 and high-risk site involvement, HDMTX was associated with a significantly lower risk of CNS relapse. The 3-year CNS relapse rates in patients who received HDMTX were 1.96% (95% CI 0.16–8.07) vs 13.7% (95% CI 8.90–35.24) without, *P* = 0.046. No significant difference in PFS or OS was found between the two groups. The 3-year PFS and OS rates were 72.7% (95% CI 56.8–83.6) and 76.9% (95% CI 59.6–87.5) in patients who received HDMTX, and 65.3% (95% CI 36.6–83.47) and 69.2% (95% CI 34.5–88.0) for patients who did not receive HDMTX respectively (*P* = 0.79 for PFS; *P* = 0.23 for OS).

### Toxicity

Toxicity data were collected for a total of 194 cycles of HDMTX given to 66 patients. The overall rate of renal toxicity was 0.4% (8/194) (one grade IV) and hepatic toxicity was 5% (9/194) (all grade I-II). Febrile neutropenia occurred in 2% of cycles (4/194) and a delay in RCHOP occurred in 3% of cycles (5/194).

## Discussion

The role of HDMTX as CNS prophylaxis and its effect on survival outcomes in patients with DLBCL at high risk for CNS relapse is contentious. Comparison between available studies is difficult due to heterogeneity in selection criteria for CNS prophylaxis, treatment regimens, and no reliable differentiation between isolated CNS relapse and CNS with concomitant systemic relapse. To the authors’ knowledge, this multicentre retrospective analysis of 226 patients is the first of its kind that specifically assesses the role of HDMTX in isolated CNS, concomitant CNS-systemic relapse, and systemic relapse in a uniform group of patients who received RCHOP, and CNS prophylaxis based on CNS-IPI score and involvement of high-risk anatomical sites. We further utilized propensity score matching and multivariable regression analysis to reduce treatment selection bias. We demonstrated that the addition of HDMTX remained an independent factor for preventing isolated CNS relapse, with 3-year relapse rates of 1.96% and 13.7%, respectively (*P* = 0.046) in the propensity score-matched patients and 3.1% and 14.6%, respectively (*P* = 0.032*)* in the unweighted cohort. However, systemic relapse rates were not significantly different between groups, which led to no significant difference in PFS and OS rates. Notably, patients with concomitant CNS-systemic relapse, which can affect 45–55% of patients with CNS relapse [17], did not benefit from HDMTX prophylaxis and had poor outcomes with median OS ~30 days in our study. Further characterization of risk factors and intensification of systemic treatment may improve outcomes in these patients, and this remains an important area of unmet need.

Methotrexate given in high doses systemically penetrates into brain tissue remote from the CSF and is highly efficacious in the treatment of primary CNS lymphoma [18]. HDMTX prophylaxis was first supported by a randomized trial showing fewer CNS recurrences with ACVBP and 2 cycles of HDMTX compared with CHOP chemotherapy (0.8% vs 2.7%, *P* = 0.02) [19]. Various retrospective or small prospective series also reported significant reductions in risk of CNS relapse with HDMTX, but either lacked a comparator arm [6, 20], or had imbalance in risk characteristics between HDMTX and control patients [7], limiting data interpretation. Recent observational studies have attempted to improve on previous studies which tended to be limited by small sample sizes and confounding factors. One of the largest analyses to date with 326 patients at high risk of CNS relapse did not find HDMTX effective, but most of the CNS relapses were concomitant CNS-systemic relapses (59% of cases) rather than isolated CNS relapses [9]. The authors found that optimizing systemic disease control with autologous transplant or intensive chemoimmunotherapy led to a significantly lower rate of CNS relapse, adding support to the hypothesis that improved systemic control of the disease is perhaps more important to reduce concurrent CNS-systemic relapse than HDMTX. Another study in an intention-to-treat analysis incorporating propensity score matching, also did not find differences in CNS relapse rate with HDMTX, but the nature of CNS relapse (whether concomitant with systemic relapse) was unclear, and patients included in study had significant heterogeneity in CNS prophylaxis indication, including raised LDH with one extranodal site involvement and HIV lymphoma. Other studies failing to show efficacy of HDMTX prophylaxis were either conducted prior to risk assessment using CNS-IPI score, had imbalance in baseline risk factors and treatment factors, or had low incidences of CNS events making it difficult to draw conclusions [14, 15]. Our results are in line with a recent study examining the impact of HDMTX on isolated CNS relapse, which found that 5-year isolated CNS relapse rates were 5% in the HDMTX group versus 26% in the group without prophylaxis [21].

In contrast to isolated CNS relapse, HDMTX did not lead to lower risk of concomitant CNS and systemic relapses in our study. We postulate that HDMTX may help to prevent dissemination of DLBCL into the CNS or prevent occult microscopic disease at diagnosis from manifesting. However, concurrent CNS and systemic relapse/progression likely represents suboptimal systemic control with subsequent acquisition of CNS penetrating malignant clones and seeding into the CNS. In these cases, more intensive chemoimmunotherapy, novel agents, or consolidation autologous transplant may be required to improve long-term control. Challenges remain regarding how to accurately predict CNS outcomes and individualize initial treatment.

While HDMTX was associated with reduced CNS relapse rates, it did not remain an independent prognostic factor for PFS or OS in multivariable analysis of the unmatched cohort or in propensity score matched analysis, likely due to its inability to reduce the risk of systemic relapse. These results contrast with some studies suggesting that HDMTX has efficacy in systemic disease and can improve patient outcomes regardless of its effects on CNS relapse. Melen et al. reported findings from a Swedish registry that patients who received cytarabine and HDMTX in addition to RCHOP had improved OS outcomes, and postulated that HDMTX may protect from systemic relapse [22], although the improved treatment effect may also be related to cytarabine. Similarly, Goldschmmidt et al. and Lee et al. found similar or better survival outcomes with HDMTX irrespective of CNS relapse rates. However, both studies did not differentiate between isolated versus concurrent CNS-systemic relapse and had heterogenous baseline risk and treatment factors (particularly rituximab in the former study). Furthermore, patients who had poor ECOG likely did not receive HDMTX which may have led to worse survival outcomes. Our results are in line with a study using intent-to-treat analysis [8], which showed no difference in survival outcomes with HDMTX. Prospective evaluation is required to determine whether intensification of RCHOP with HDMTX can improve survival.

HDMTX requires inpatient administration and is associated with adverse side effects such as nephrotoxicity, hepatotoxicity, and myelosuppression. Our institution delivers HDMTX at 3 g/m^2^ by day 3 of RCHOP and noted a low rate of toxicity including elevated creatinine (4%), raised transaminases (5%), and neutropenic fever (2%). Grade III/IV adverse events were rare. Importantly, only 2.5% of the RCHOP cycles were delayed using this schedule. HDMTX with concurrent RCHOP can be administered safely, and renal dysfunction was uncommon and self-limited with careful attention to adequate hydration and urine alkalinisation. While the incidence of early CNS events forms the rationale for introduction of CNS directed therapy concurrently with systemic induction [23], this must be balanced with risks of toxicity for the individual patient.

This study has several limitations. The major limitation lies in its retrospective nature; prospective validation is warranted. However, we performed propensity score matched analyses to overcome potential bias between groups. We also included only DLBCL patients who received RCHOP chemotherapy with uniform selection criteria for CNS prophylaxis based on CNS-IPI score and high-risk site involvement. The small number of concurrent CNS and systemic relapse events is another limitation of this study, potentially restricting the power of analysis. Thirdly, it was not routine practice to perform lumbar punctures at the time of DLBCL diagnosis in the absence of neurologic signs or symptoms in the past, and this was done at the discretion of the treating physician. While we excluded patients with isolated CNS relapse occurring within 4 months of treatment, as this may suggest synchronous involvement at diagnosis, there is still a chance that some patients have occult CNS disease. However, the estimated CNS relapse rates and their 95% CIs in this study are consistent with previous studies including the original CNS-IPI study [1], suggesting that data is representative. Lastly, the relatively shorter follow-up duration may underestimate late relapses [24].

In conclusion, HDMTX prophylaxis was associated with reduced isolated CNS relapse rates but not systemic or concurrent CNS-systemic relapse, with no difference in survival outcomes in our study. Toxicity was manageable when delivered by day 3 of RCHOP. Our study suggests that effective prophylaxis against isolated CNS relapse can be provided with HDMTX in a high-risk cohort, possibly by treating occult microscopic disease or preventing dissemination during primary therapy. Further investigation is necessary to better define the group of patients who will benefit from HDMTX prophylaxis, and the optimal dose and timing, while minimizing potential toxicities.

## Supplementary information

Supplementary Material
