# Supplementary material for: High-dose methotrexate is effective for prevention of isolated CNS relapse in diffuse large B cell lymphoma
Source: Blood Cancer J. 2021 Aug 12;11(8):143. doi: 10.1038/s41408-021-00535-y (PMC8361130; doi:10.1038/s41408-021-00535-y)
Supplement: Supplementary file 1 — Supplementary Material [file 41408_2021_535_MOESM1_ESM.docx]

**Supplementary Table 1.**Univariable analysis of CNS relapse and systemic by high dose methotrexate prophylaxis

|  | CNS relapse (isolated and with systemic) vs disease free | | Systemic relapse vs disease free | |
| --- | --- | --- | --- | --- |
|  | HR (95% CI) | *P*value | HR (95% CI) | *P*value |
| Age (<60 vs ≥60) | 1.25 (0.58-2.68), | 0.565 | 1.19 (0.61-2.31) | 0.616 |
| Gender (male vs female) | 0.80 (0.40-1.59), | 0.525 | 0.97 (0.53-1.77) | 0.930 |
| Stage 3 or 4, n (%) | 2.87 (0.39-21.0), | 0.30 | 3.24 (0.45-23.54) | 0.246 |
| Elevated LDH | 1.27 (0.39-4.15), | 0.696 | 1.10 (0.39-3.08) | 0.859 |
| EN site >1  EN site involvement    Bone marrow    Kidney/adrenal    Testis    Breast | 3.86 (0.92-16.13)    **3.23 (1.60-6.54)**  1.77 (0.89-3.50)  **5.15 (1.81-14.69)**  **7.88 (1.87-33.17)** | **0.064**    **0.001**  0.103  **0.002**  **0.005** | **3.88 (0.94-16.08)**    1.28 (0.69-2.35)  0.59 (0.26-1.32)  1.70 (0.23-12.41)  3.29 (0.45-24.01) | **0.061**    0.435  0.200  0.601  0.241 |
| Cell-of-origin (GCB) | 1.09 (0.53-2.29) | 0.818 | 1.50 (0.80-2.82) | 0.208 |
| IPI 3-5 vs 0-2 | 2.65 (0.63-11.05) | 0.182 | **5.85 (0.80-42.51)** | **0.081** |
| CNS-IPI 4-6 vs 0-3 | 3.34 (0.80-13.96) | **0.098** | 1.73 (0.62-4.84) | 0.300 |
| HDMTX | **0.22 (0.07-0.72)** | **0.012** | 0.82 (0.40-1.70) | 0.598 |
| IT methotrexate | 0.89 (0.39-2.05) | 0.786 | 0.49 (0.19-1.24) | 0.133 |

LDH, lactate dehydrogenase; GCB, germinal center B-cell like; EN, extranodal; IT, intrathecal; HDMTX, high dose methotrexate

**Supplementary table 2.** Propensity score-matched baseline characteristics

|  | No HDMTX (n=51) | HDMTX (n=51) | SMD |
| --- | --- | --- | --- |
| LDH | 47 (92.2%) | 47 (92.2%) | 0.000 |
| Stage 3 or 4 | 47 (92.2%) | 49 (96.1%) | 0.017 |
| EN>1  EN site involvement  Testis  Bone marrow  Kidney/adrenal  Breast | 37 (72.5%)    2 (3.9%)  26 (51.0%)  12 (23.5%)  1 (1.9%) | 39 (76.4%)    2 (3.9%)  23 (45.1%)  14 (27.5%)  1 (2.0%) | 0.089    0.000  0.117  0.089  0.000 |
| CNS-IPI 4-6 | 42 (82.4%) | 42 (82.4%) | 0.000 |

Data are n (%), unless otherwise stated. LDH, lactate dehydrogenase; EN, extranodal; SMD, standardized mean difference.
